# Osteitis fibrosa cystica mimicking bone tumor, a case report

**DOI:** 10.1186/s12891-021-04374-7

**Published:** 2021-05-25

**Authors:** Maya L. Nasser, Serge Medawar, Tonine Younan, Halim Abboud, Viviane Trak-Smayra

**Affiliations:** 1 Pathology Department, Hotel-Dieu de France Hospital, Saint Joseph University Medical School, PO Box 16-6830, Alfred Naccache Bvd, Ashrafieh, Beirut, Lebanon; 2 Radiology Department, Hotel-Dieu de France Hospital, Saint Joseph University Medical School, Beirut, Lebanon; 3 Neurology Department, Hotel-Dieu de France Hospital, Saint Joseph University Medical School, Beirut, Lebanon

**Keywords:** Hyperparathyroidism, Hypercalcemia, Osteitis fibrosa cystica, Brown tumor, Case report

## Abstract

**Background:**

We report a case of osteitis fibrosa cystica, a rare benign resorptive bone lesion caused by hyperparathyroidism, that presented on imaging as an aggressive bone tumor.

**Case presentation:**

The patient is a 51-year-old male complaining of severe sustained pain of the right hip region. Imaging studies were suspicious for a malignant tumor of the right iliac bone. Biopsy under CT guidance was performed and showed remodeled bone trabeculae with numerous osteoclasts, excluding bone tumor and raising the possibility of osteitis fibrosa cystica. Complementary tests disclosed elevated blood level of parathyroid hormone and a partially cystic enlarged left inferior parathyroid gland consistent with adenoma. After parathyroidectomy, the clinical symptoms were relieved and the radiological findings were significantly improved, which confirmed the diagnosis.

**Conclusions:**

Metabolic diseases-associated bone lesions should always be considered in the differential diagnosis of bone tumors, to avoid unnecessary surgeries and treatments.

## Background

Primary hyperparathyroidism is a common endocrine disorder characterized by elevated blood concentration of parathyroid hormone (PTH) and hypercalcemia, usually due to a benign overgrowth of parathyroid tissue. It predominantly affects women, especially after menopause [1]. Primary hyperparathyroidism is often asymptomatic, detected by routine biochemical screening. However, severe forms may be revealed by lesions of target organs such as the skeletal system and the kidneys. Among patients with primary hyperparathyroidism, less than 20 % develop overt kidney stone disease [2]. Radiologically evident bone disease is even less common, and manifests as osteopenia, subchondral and subperiosteal bone resorption, acro-osteolysis or rarely brown tumors (BT)[2, 3]. In fact, the most common skeletal manifestation of primary hyperparathyroidism is loss of cortical bone due to osteoclast activation with relatively preserved trabecular bone. More severe syndrome of skeletal loss is seen in osteitis fibrosa cystica (OFC). OFC can be associated clinically with fractures, skeletal deformities and bone pain and manifests as bone erosions, bone resorption, BT and cysts [1, 3]. We report a case of primary hyperparathyroidism with OFC that presented clinically and radiologically as an aggressive bone tumor.

## Case presentation

A 51-year-old male presented to our institution for pain in the right hip irradiating to the lower limb. The pain was persisting at rest, and sometimes worsened with movement, especially during the last 6 months before admission. He is a known smoker but did not report any chronic diseases. The patient reported a history of trauma to the lumbar spinal region secondary to a fall, three years ago treated medically. Physical examination was unremarkable and excluded any neurologic deficit that could account for his pain.

**Fig. 1:**
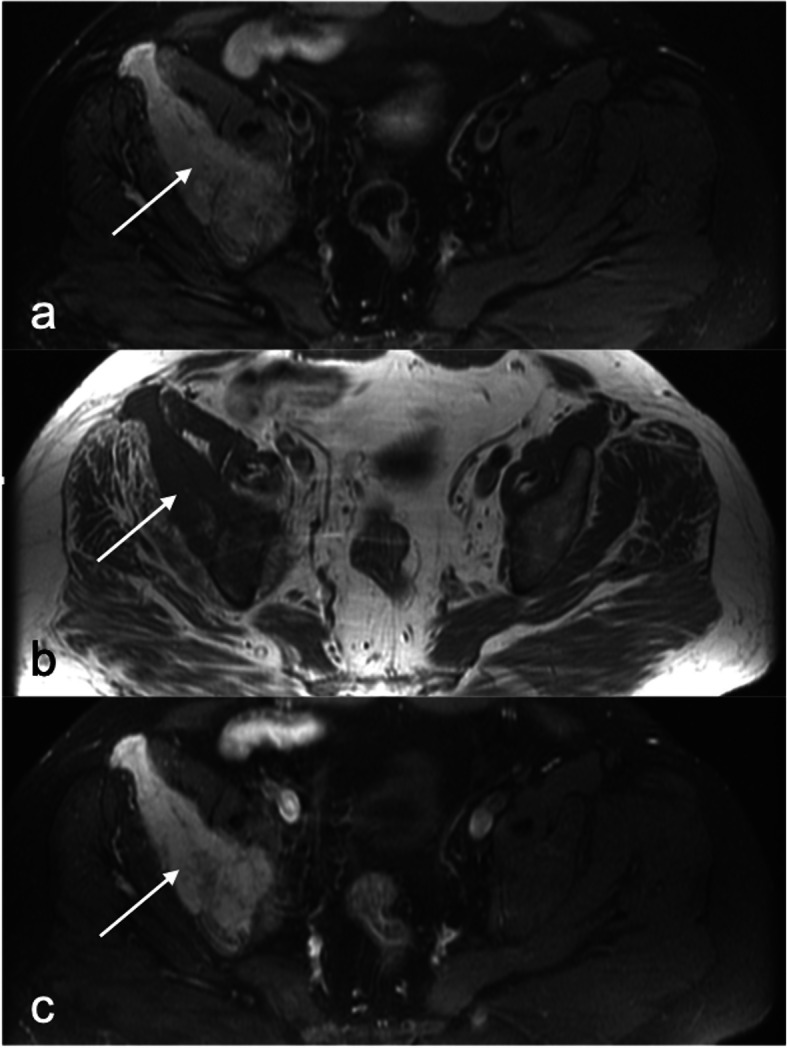
Magnetic resonance imaging of the pelvis showing the expansile and destructive lesion affecting the right iliac bone (arrows), hyperintense on proton density Fat Sat weighted image (**a**), of intermediate signal with some normal bony streaks on T1 weighted image (**b**) and showing extensive contrast enhancement after IV injection of gadolinium (**c**)

**Fig. 2:**
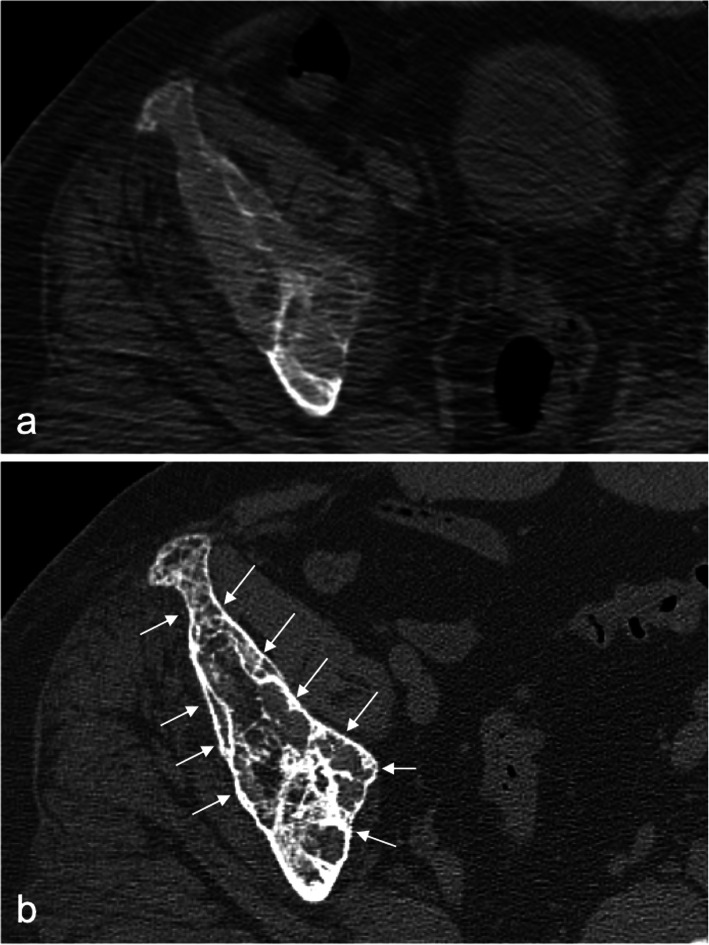
Initial single-row detector computed tomography (CT) of the pelvis showing extensive lytic lesion, affecting the entire right iliac bone, with destruction of bone cortices and the presence of some normal bony streaks. This lesion is spontaneously hyperdense (130 UH). There is also diffuse bone enlargement without the coarsened trabecular pattern found in Paget disease (**a**). Ten months after parathyroidectomy (**b**), CT shows advanced healing of the lytic lesion of the right iliac bone with complete reconstruction of bone cortices (small arrows) and formation of intra medullary, thickened bone septa

**Fig. 3:**
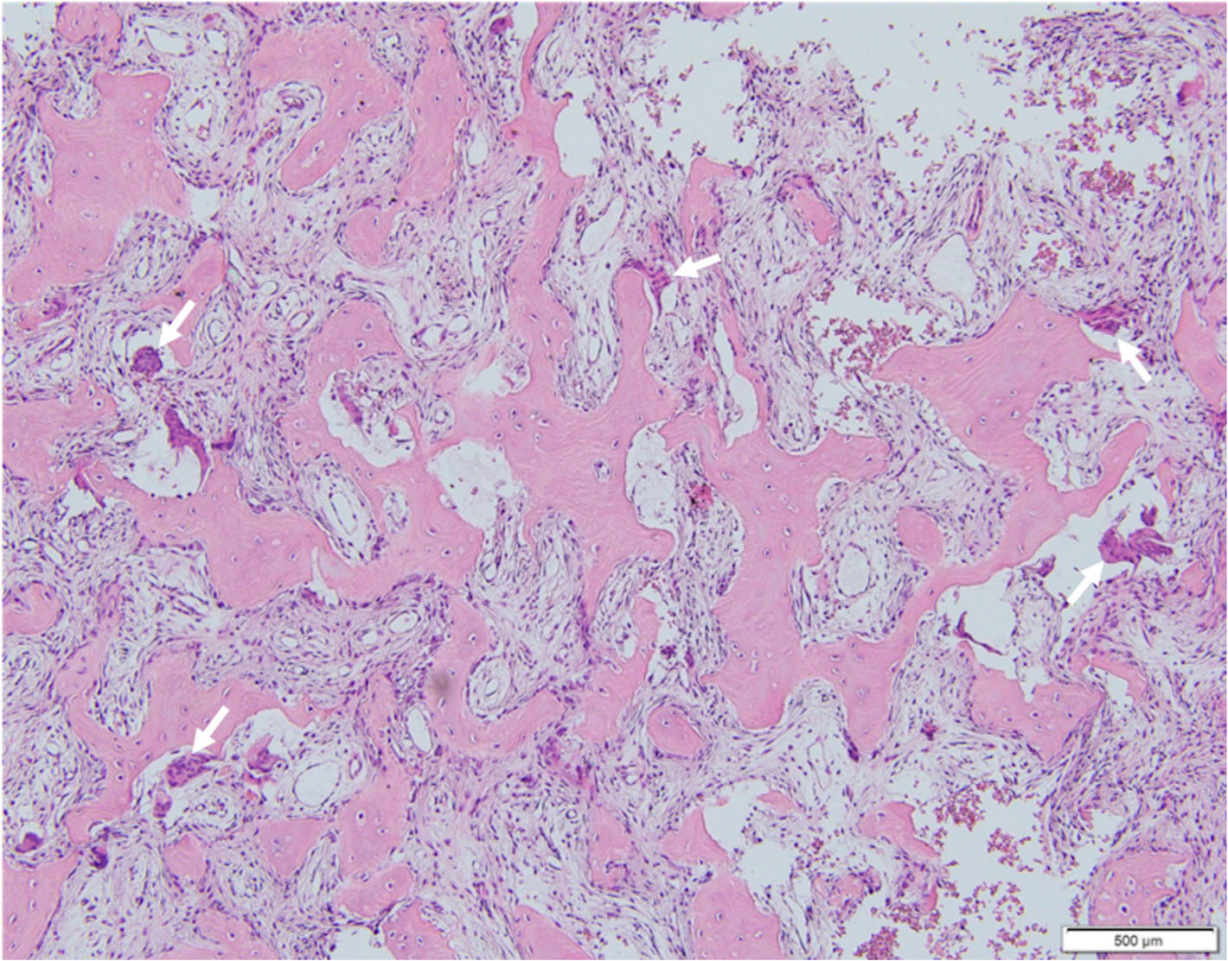
Microscopic examination of the right iliac bone biopsy showing remodeled bone trabeculae within fibrovascular tissue and unusual high number of osteoclasts (arrows) (H&E x 100)

**Fig. 4:**
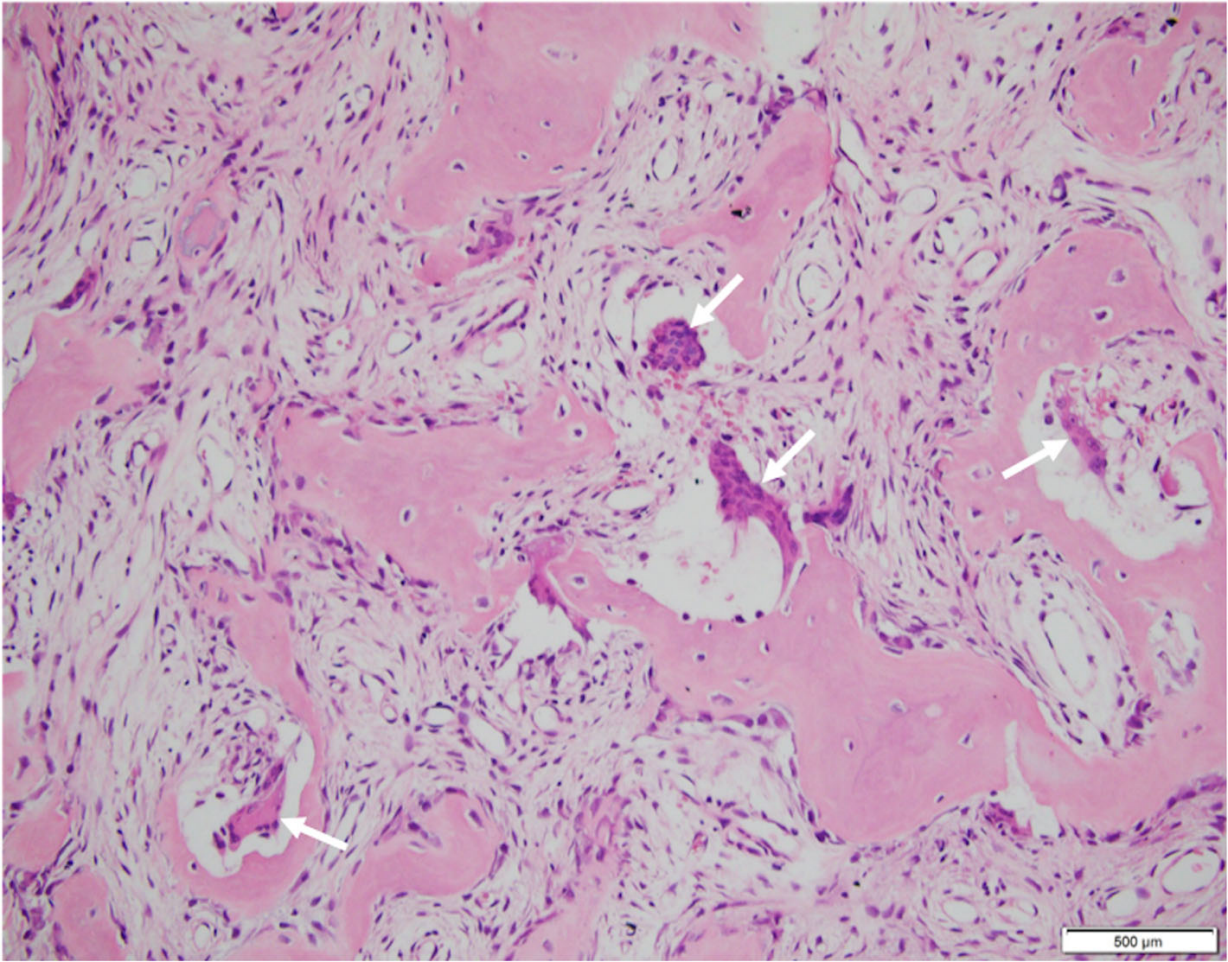
On higher magnification, several Howship’s lacunae are seen, formed by osteoclasts tunneling into or dissecting bone trabeculae (arrows) (H&E x 200)

MRI of the pelvic bones showed a diffuse fleshy intraosseous lesion involving the entire right iliac bone extending from the iliac crest to the superior ramus and ischial ramus and the entire right acetabulum. This lesion appeared to extend into the adjacent soft tissues, mainly in the iliopsoas and obturator muscles (Fig. 1). A suspicious pathologic fissure was noted in the ischial ramus. The right sacral bone showed features of edema. The MRI findings were suggestive of lymphoma or metastasis. A primary bone tumor could however not be excluded. CT scan, performed 3 days after the MRI, showed extensive lytic lesion, affecting the entire right iliac and ischial bones, with destruction of bone cortices and the presence of some normal bony streaks. This lesion was spontaneously hyperdense (130 UH). There was also diffuse bone enlargement without the coarsened trabecular pattern usually found in Paget disease (Fig. 2a). Biochemical blood tests revealed low phosphorus concentration (2,3 mg/dL, normal values 2,5–5 mg/dL) and high calcium concentration (11.3 mg/dL, normal values 8,4–10,5 mg/dL). These findings were deemed secondary to the bone tumor. A bone biopsy was performed under CT scan guidance, several cores were sampled and sent for culture and histopathologic examination. Culture returned negative results, ruling out the possibility of infection. Histopathologic evaluation showed remodeled and newly formed trabecula of cancellous bone separated by a cellular fibrovascular, edematous tissue containing several osteoclasts (Fig. 3). Characteristically the osteoclasts were tunneling into bone trabeculae forming Howship’s lacunae (Fig. 4). There was no evidence of malignancy in the biopsy sample. The histopathologic findings were suggestive of OFC associated with hyperparathyroidism. PTH dosage was subsequently performed, showing high blood level (389.6 pg/ml, normal values 10–69 pg/ml). Cervical ultrasound revealed a 52 × 22 mm mixed solid and cystic nodule in the left inferior posterior pole of the thyroid gland, consistent with a parathyroid cystic adenoma.

After parathyroidectomy, the patient no longer complained from bone pain and imaging performed few months later showed significant improvement, with reduction of the bone lesion dimensions and complete reconstruction of bone cortices (Fig. 2b).

## Discussion and conclusion

Bone lesions related to hyperparathyroidism are often diagnosed late because they are clinically silent unless the disease reaches advanced stages and causes clinical symptoms such as pain or deformity. They may also be misdiagnosed because of their similarity to many other bone diseases and malignancies and because of their rare occurrence in contemporary practice. OFC is seen in almost 5 % of patients with primary hyperparathyroidism on presentation [4]. This is explained by the fact that, in many developed countries, laboratory screening tests are available, allowing the detection of asymptomatic hyperparathyroidism. Skeletal manifestations of primary hyperparathyroidism have become uncommon and thus, not considered in the differential diagnosis. Our patient comes from a disadvantaged socioeconomic background, and from a region with poor healthcare infrastructure. These factors can explain undiagnosed hyperparathyroidism that progressed to worsening bone pain and severe lytic bone lesions on imaging.

OFC is diagnosed through radiological features such as “salt-and-pepper” degranulation of the skull, tapering of the distal clavicle, subperiosteal resorption of the radial aspects of the proximal and middle phalanges of the second and third fingers, bone cysts, and BT [2, 5]. Also, there is a generalized decrease in bone mineral density, preferentially affecting cortical bone over cancellous bone. However, the radiological features of other lesions affecting the bone, especially malignant tumors, such as metastatic carcinoma, osteosarcoma, Ewing sarcoma, lymphoma and multiple myeloma may be very similar to bone lesions associated with hyperparathyroidism and can be considered in the differential diagnosis. Symptomatic hypercalcemia may accompany such lesions and be interpreted as a manifestation of the malignancy itself [6]. To be able to differentiate between hypercalcemia induced by primary hyperparathyroidism and that secondary to malignancy, the blood level of PTH, which should be significantly elevated in hyperparathyroidism, is the clue to diagnosis. In the hypercalcemias of malignancy, high PTH concentrations are virtually never seen [6].

Another differential diagnosis to ponder when considering such bone lesions is osteomyelitis, which was ruled out in our case by biopsy and microbiological analysis.

Biopsy helped also excluding a malignant tumor and showed bone remodeling and conspicuous bone resorption by osteoclasts, suggesting a metabolic bone lesion related to hyperparathyroidism, which was, later on, confirmed by a high blood level of PTH and the finding of a parathyroid gland adenoma on neck ultrasound. Microscopically, bone lesions associated with hyperparathyroidism usually demonstrate increased osteoclast and osteoblast activity, bone turnover, with marked resorption and new bone formation complemented by peritrabecular fibrosis of the adjacent marrow. In more advanced stages, degree of remodeling is high and aggressive osteoclast activity results in thinned cortices. Moreover, cancellous bone trabeculae can undergo tunneling resorption where the trabeculae split [7]. In severe cases, BT develops. BT which was not detected in the case presented here is often a manifestation of OFC and may be histologically confused with giant cell tumor (GCT), giant cell reparative granuloma (GCRG) and solid variant of aneurysmal bone cyst (SV-ABC) [6]. BT is a non-neoplastic reactive lesion associated with hyperparathyroidism. It has a typical brownish shade due to deposits of hemorrhage and hemosiderin. GCT is a benign tumor characterized by an excess of multinuclear giant cells exhibiting the features of mature osteoclasts along with stromal round mononuclear cells. BT and GCT are similar in that they are both composed of mononuclear cells mixed with multinuclear giant cells. However, it was reported that giant cells tend to be distributed uniformly in GCT, whereas in BT they are usually arranged in clusters [8]. On the other hand, BT has much more lobulated architectural growth pattern than GCRG or SV-ABC. However, these lesions may be indistinguishable on small biopsy samples [9]. Paget’s disease is a chronic skeletal disorder of abnormal bone remodeling affecting mainly older adults [7]. In its early course, Paget’s disease is detected radiographically by osteolytic, radiolucent lesions, and histologically by elevated osteoclast and osteoblast activity and marrow fibrosis that is similar to hyperparathyroidism [7]. Distinction between the two entities is difficult in small biopsies. However, in our case, the discovery of elevated blood PTH and the clinical and radiological improvement after parathyroidectomy allowed to confirm the diagnosis of OFC.

The diagnosis of OFC may be challenging. In addition to the histological and radiological findings, biochemical screening and in-depth medical history are very important to ensure a correct OFC diagnosis. Post-surgical outcomes are important as well. In the case of primary hyperparathyroidism, parathyroidectomy usually results in a halt of bone deterioration, improved bone mineral density and reduced risk of fracture, as well as regression of BT, when present [10]. In the absence of post-surgical amelioration, the possibility of concomitant bone diseases should be considered.

In summary, metabolic diseases-associated bone lesions can mimic bone tumors and should always be considered in the differential diagnosis of patients presenting for bone tumors, to avoid unnecessary surgeries and treatments. In the rare cases where primary hyperparathyroidism is symptomatic at the skeletal level, it usually presents as OFC. Elevated PTH levels and hypercalcemia are indicative of this disease and complement the radiological and histological features to confirm the diagnosis. Parathyroidectomy usually results in halt of bone deterioration and improved bone lesions.

## Data Availability

The authors declare that data supporting the findings of this study are available within the article.

## Abbreviations

BT: Brown tumor
GCRG: Giant cell reparative granuloma
GCT: Giant cell tumor
OFC: Osteitis fibrosa cystica
PTH: Parathyroid hormone
SV-ABC: Solid variant of aneurysmal bone cyst
